# Immigration and access to dementia diagnostics and treatment: A nationwide study in Sweden

**DOI:** 10.1016/j.ssmph.2023.101573

**Published:** 2023-12-02

**Authors:** Minh Tuan Hoang, Ingemar Kåreholt, Emma Lindgren, Lena von Koch, Hong Xu, Edwin C.K. Tan, Kristina Johnell, Katarina Nägga, Maria Eriksdotter, Sara Garcia-Ptacek

**Affiliations:** a Division of Clinical Geriatrics, Department of Neurobiology, Care Sciences and Society, Karolinska Institutet, Stockholm, Sweden; b Institute of Gerontology, School of Health Welfare, Aging Research Network – Jönköping (ARN-J), Jönköping University, Jönköping, Sweden; c Aging Research Center (ARC), Karolinska Institutet and Stockholm University, Stockholm, Sweden; d Memory Clinic, Skåne University Hospital, Malmö, Sweden; e Division of Family Medicine and Primary Care, Department of Neurobiology, Care Sciences and Society, Karolinska Institutet, Stockholm, Sweden; f Neuro Theme, Karolinska University Hospital, Stockholm, Sweden; g Faculty of Medicine and Health, School of Pharmacy, The University of Sydney, New South Wales, Australia; h Department of Medical Epidemiology and Biostatistics, Karolinska Institutet, Stockholm, Sweden; i Clinical Memory Research Unit, Department of Clinical Sciences Malmö, Lund University, Malmö, Sweden; j Department of Acute Internal Medicine and Geriatrics, Linköping University, Linköping, Sweden; k Theme Inflammation and Aging, Karolinska University Hospital, Stockholm, Sweden

**Keywords:** Dementia, Diagnosis, Difference, Drug, Ethnic, Inequality, Treatment

## Abstract

• Compared to Swedish-born people, foreign-born people were less likely to receive dementia diagnostic tests.
• Being born in Africa or Europe was associated with lower chance of receiving cholinesterase inhibitors.
• Asian-born people had higher chance of receiving cholinesterase inhibitors, but were less likely to receive memantine.
• Disparities existed in dementia diagnostics and treatment between Swedish-born and foreign-born people, but were not consistent after adjusting for MMSE scores.

## 1 Introduction

Health inequality is defined as any difference in health status or in the allocation of health resources among groups of people or countries (Whitehead, 1992). The World Health Organization states that public health responses to dementia must protect the health equality for the vulnerable, including immigrants (World Health Organization, 2017). Immigration is an important determinant of health. Assessing the disparities in healthcare services for persons with dementia was crucial, especially when inequalities in dementia care related to education, income, or sex were shown in previous studies (Garcia-Ptacek et al., 2017; Hoang et al., 2020, 2021; Roheger, Eriksdotter, Westling, Kalbe, & Garcia-Ptacek, 2019). All suspected dementia cases should be identified early because this helps persons with dementia prepare their finances, living conditions, and gives them access to treatment (Alzheimer's Disease International, 2011; Dubois, Padovani, Scheltens, Rossi, & Dell'Agnello, 2016; World Health Organization, 2017).

Previous studies showed ethnic inequalities in dementia care (Hinton, Franz, & Friend, 2004; Nielsen, Vogel, Phung, Gade, & Waldemar, 2011), and delay in access to diagnosis among immigrants (Barker et al., 2005; Segers et al., 2013). A study in Norway found immigrants from low- and middle-income countries had lower chances to receive dementia diagnosis compared to Norwegian-born people (Diaz, Kumar, & Engedal, 2015). A recent study in the USA mentioned that non-Hispanic, Blacks and Hispanics underwent missed or delayed diagnoses of dementia more frequently than non-Hispanic Whites (Lin et al., 2021). Another study observed lower chances of receiving anti-dementia drugs and residing in a nursing home for Western and non-Western immigrants with dementia, compared to Danish-born persons with dementia (Stevnsborg, Jensen-Dahm, Nielsen, Gasse, & Waldemar, 2016). Other studies also showed immigrants were less likely to receive anti-dementia drugs compared to native persons with dementia (Cooper, Tandy, Balamurali, & Livingston, 2010; Mehta, Yin, Resendez, & Yaffe, 2005; Poon, Lal, Ford, & Braun, 2009; Zuckerman et al., 2008).

In Sweden, immigrants constitute a growing proportion of the population, with over two million foreign-born people by 2020 (about one-fifth of total population) (Statistics Sweden, 2020). A recent study revealed that foreign-born persons with dementia were less likely to receive a specific dementia diagnosis or cholinesterase inhibitors compared to Swedish-born people (Lindgren, Sörenson, Wattmo, Kåreholt, & Nägga, 2021). This study compared persons with dementia aged 65 and above who were born in Sweden with those born in the high-, middle- and low-income countries (Lindgren et al., 2021).

In this nationwide study, we aimed to investigate the disparities in dementia diagnostic process and the prescription of anti-dementia drugs between Swedish-born and foreign-born persons with dementia. We hypothesized that foreign-born people were less likely to receive dementia diagnostic tests and anti-dementia drugs, compared to Swedish-born people.

## 2 Materials and methods

Reporting was in accordance with the REporting of studies Conducted using Observational Routinely collected health Data (RECORD) statement (Benchimol et al., 2015) (Supplementary eTable 1). This study was approved by the Swedish Ethical Review Authority (decision number 2017/501–31; 2017/1448–32; 2021–05289).

### 2.1 Study design and setting

In this observational study, we used data of persons with dementia registered in the Swedish registry for cognitive/dementia disorders - SveDem between 2007 and 2018. Created in 2007, SveDem is a national quality of care register, including persons with dementia in Sweden who are diagnosed according to the International Classification of Diseases, Tenth Revision (Religa et al., 2015). Data of persons with dementia were linked with other Swedish national registries via the Swedish personal identification number (the personal identification of persons with dementia was pseudonymized and blinded before delivering to researchers). Founded in 1990, the Swedish Longitudinal Integrated Database for Health Insurance and Labor Market Studies - LISA records information regarding education, employment, and income of all people over 15 years old (Ludvigsson, Svedberg, Olen, Bruze, & Neovius, 2019). The Swedish National Patient Register collects data from hospitalizations and specialist diagnoses (Ludvigsson et al., 2011). The Swedish Prescribed Drug Register contains all dispensed prescribed drugs in pharmacy in Sweden (Wallerstedt, Wettermark, & Hoffmann, 2016).

### 2.2 Participants

**Fig. 1:**
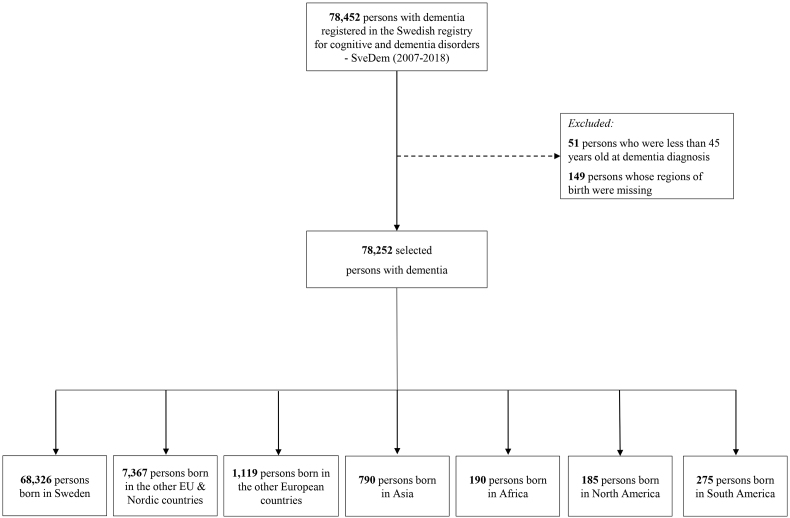
Participant selection chart.

The process of selecting participants was illustrated in Fig. 1. A total of 78,452 people were diagnosed with dementia from 2007 to 2018 and registered in SveDem. Inclusion criteria were (1) the regions of birth of participants was not missing, and (2) the age of dementia diagnosis was 45 years old or above. Dementia is a neurodegenerative condition that normally affects older individuals. Dementia is rare under age 55. To be conservative and not unnecessarily exclude cases of dementia, but also to avoid false diagnoses from neurodevelopmental disorders, we arbitrarily put the cut at age 45. Finally, 78,252 persons with dementia were retained for the main analysis.

### 2.3 Variables & data sources

Region of birth, extracted from LISA, was the main exposure. Swedish-born persons with dementia were compared with those born in the other EU and Nordic countries, the other European countries, Asia, Africa, North America, and South America. A child born in foreign countries, but to a Swedish citizen is regarded as born in Sweden, as are children of immigrants who are born in Sweden (Ludvigsson et al., 2019).

Covariates retrieved from LISA included education, income, living alone and living areas (urban, intermediate vs. rural). Education, which was the highest educational attainment, was divided into four categories: compulsory education (primary school and secondary school (years 1–9)), upper secondary (high school (years 10–12)), university (college, university or higher), and unknown. Education of immigrants was explored by annual questionnaires. If immigrants participate in any educational activity in Sweden, the new level of education will be recorded and override the older one, via their personal identity number. Annual individual income was defined as the total income that a person received after paying taxes (including all types of income such as salary, allowances, pension and etc). Income of persons with dementia one year before dementia diagnosis was inflated into 2022 values with inflation rate from the Swedish Consumer Price Index (Statistics Sweden, 2023). The inflated income from 64,848 SEK was then trichotomized into three equal groups. This threshold is normally the lowest possible income because lower incomes or pensions are complemented up to this level with universal government support (The Swedish Pensions Agency, 2023). People with incomes lower than this amount were assumed to live off other assets or savings which would have disqualified them from receiving the minimum financial support. Thus, the inflated incomes that were less than 64,848 SEK or negative were categorized as unknown. Other covariates, extracted from SveDem, encompassed age at dementia diagnosis, sex, type of diagnostic unit (primary care vs. memory clinic) and year of dementia diagnosis. In 2010, the Swedish National Board of Health and Welfare released the national guidelines on dementia care (The Swedish National Board of Health and Welfare, 2017). Hence, we categorized the year of dementia diagnosis into two periods: from 2007 to 2010, and from 2011 to 2018 because the application of the national guidelines might affect the dementia diagnostics and the use of anti-dementia drugs. Comorbidities before dementia diagnosis, extracted from the Swedish National Patient Register, were converted into Charlson Comorbidity Index (Ludvigsson et al., 2021) (Supplementary Table 2). Drug utilization during one year before dementia diagnosis was retrieved from the Swedish Prescribed Drug Register (Supplementary Table 3).

Outcomes included dementia diagnostics and the use of anti-dementia drugs. The basic dementia diagnostic work-up, which is recommended by the Swedish Board of Health and Welfare (The Swedish National Board of Health and Welfare, 2017), is an important quality indicator in SveDem. It includes the completion of four tests: clock test, blood analysis, Mini-Mental State Examination (MMSE) and computed tomography or magnetic resonance imaging (CT-MRI). An interview with persons with dementia and a knowledgeable person close to them, and a physical and psychiatric medical examination are also part of the basic work-up, but not specifically recorded in SveDem. Other individual tests included in this study were neuropsychological assessment, occupational therapy assessment, and lumbar puncture. Cholinesterase inhibitors (donepezil, galantamine and rivastigmine), and memantine are the anti-dementia drugs currently approved to use in Sweden.

### 2.4 Statistical analyses

Numerical variables were described with median and interquartile range (IQR). Categorical variables were displayed as number of cases and percentages. Information about dementia diagnostics and the prescription of anti-dementia drugs was recorded at one time point as performed/not performed for the diagnostic items and prescribed/not prescribed for the drugs. With dichotomous outcomes, binary logistic regressions are one of the suitable regression models. We used binary logistic regression to examine the association between each outcome and region of birth. In the model 1, dementia diagnostics and the use of anti-dementia drugs were regressed on regions of birth and unadjusted. Model 2 was adjusted for age at dementia diagnosis and sex. Model 3 was additionally adjusted for living alone, living areas, education, income, Charlson Comorbidity Index, year of diagnosis and type of diagnosis unit. In the analysis of cholinesterase inhibitors and memantine, only persons with Alzheimer's disease, mixed dementia, Lewy Body dementia or Parkinson's disease dementia were considered (n = 42,005). Post-estimation was performed with the Wald test to examine the overall association of region of birth and each outcome. The goodness of fit of regression models was evaluated with the Hosmer-Lemeshow test. Results of Hosmer-Lemeshow tests were presented for Model 2 and Model 3. The Hosmer-Lemeshow test is not suitable for models with only one independent variable that is given dummy representation. Therefore, the test is not applied for Model 1.

Subgroup analyses were conducted on sex, type of diagnostic unit and year of diagnosis. MMSE is a tool for evaluating cognitive impairment (Folstein, Folstein, & McHugh, 1975; Pezzotti, Scalmana, Mastromattei, & Di Lallo, 2008). Nonetheless, MMSE can only describe the cognitive impairment partly because age, culture, education, and language barriers can influence individual MMSE scores (Crum, Anthony, Bassett, & Folstein, 1993; Nielsen & Jørgensen, 2020). Sensitivity analysis was performed by adjusting for MMSE scores in the regression models to evaluate how this partial representation of cognitive function affected the association between region of birth and each outcome. In the first regression model, we selected people who received MMSE (n = 73,211). The second model was performed on people who both received and did not receive MMSE (n = 78,252). From our previous studies, persons who do not receive MMSE at work-up have higher mortality than people testing between 0 and 10 points (Garcia-Ptacek et al., 2014). We assumed that they mostly represented more severe cases and set their MMSE scores were 5 points.

Results were reported with odds ratios (OR) and 95% confidence interval (95% CI). The statistical analyses in this study were conducted with STATA version 17.0 (StataCorp, College Station, TX). All statistical tests were two tailed with a p-value equal or less than 0.05 considered statistically significant. Listwise deletion was applied in the regression models because of the small number of missing in both exposure and covariates (less than 1%) and missing was assumed to be at random.

## 3 Results

### 3.1 Characteristics of the study population

Most persons with dementia in this study were born in Sweden, other EU, or Nordic countries (Fig. 1 and Table 1). North American-born individuals had the highest median age, 83 years old (IQR 8.0). Meanwhile, African-born people had the lowest median age, 72 years old (IQR 14.0). Swedish-born persons with dementia had the highest proportion of living in rural areas (n = 21,153, 31.0%), compared to foreign-born individuals. The percentage with compulsory education was highest among Swedish-born persons with dementia (n = 31,221, 45.7%), and lowest among African-born individuals (n = 39, 20.5%). However, African- and Asian-born persons with dementia also had the highest percentages of unknown education, with 31.1% and 33.4%, respectively. Regarding individual income levels, Swedish-born and North American-born persons with dementia had the highest proportions of people in the highest income tertile, with 32.7% and 36.2%, respectively. Meanwhile, people born in Africa, Asia, and South America were more frequent in the lowest income category.

**Table 1:** Characteristics of dementia patients from different regions of birth (n = 78,252).

	Sweden (n = 68,326)	Other EU & Nordic countries (n = 7367)	The other European countries (n = 1119)	Asia (n = 790)	Africa (n = 190)	North America (n = 185)	South America (n = 275)
Age at dementia diagnosis, years, median (IQR)	81.0 (10.0)	80.0 (10.0)	78.0 (10.0)	76.0 (13.0)	72.0 (14.0)	83.0 (8.0)	77.0 (12.0)
*Age 45–64*	2767 (4.0)	336 (4.6)	107 (9.6)	134 (17.0)	55 (28.9)	8 (4.3)	36 (13.1)
*Aged 65–74*	13,348 (19.5)	1563 (21.2)	258 (23.1)	218 (27.6)	59 (31.1)	21 (11.4)	68 (24.7)
*Aged >74*	52,211 (76.4)	5468 (74.2)	754 (67.4)	438 (55.4)	76 (40.0)	156 (84.3)	171 (62.2)
Sex, female, n (%)	39,722 (58.1)	4729 (64.2)	642 (57.4)	415 (52.5)	94 (49.5)	93 (50.3)	177 (64.4)
Municipality types, n (%)							
*Urban*	25,211 (36.9)	3716 (50.5)	645 (57.7)	528 (66.9)	143 (75.3)	100 (54.1)	198 (72.0)
*Intermediate*	21,929 (32.1)	2234 (30.3)	351 (31.4)	230 (29.2)	32 (16.8)	56 (30.3)	59 (21.5)
*Rural*	21,153 (31.0)	1412 (19.2)	121 (10.8)	31 (3.9)	15 (7.9)	29 (15.7)	18 (6.5)
Living alone, n (%)	35,871 (53.3)	4140 (57.3)	579 (52.4)	327 (42.2)	78 (41.7)	99 (54.4)	155 (57.0)
Education							
*University*	7143 (10.5)	641 (8.7)	60 (5.4)	76 (9.6)	25 (13.2)	45 (24.3)	23 (8.4)
*Upper secondary*	29,533 (43.2)	3229 (43.8)	353 (31.5)	191 (24.2)	67 (35.3)	78 (42.2)	91 (33.1)
*Compulsory education*	31,221 (45.7)	3176 (43.1)	450 (40.2)	259 (32.8)	39 (20.5)	51 (27.6)	119 (43.3)
*Unknown*	429 (0.6)	321 (4.4)	256 (22.9)	264 (33.4)	59 (31.1)	11 (5.9)	42 (15.3)
Annual individual income							
*>194,768 SEK*	22,365 (32.7)	1764 (23.9)	138 (12.3)	68 (8.6)	34 (17.9)	67 (36.2)	31 (11.3)
*155, 005 SEK- 194,768 SEK*	21,419 (31.3)	2325 (31.6)	365 (32.6)	196 (24.8)	46 (24.2)	44 (23.8)	76 (27.6)
*64,848 SEK-155, 005 SEK*	20,649 (30.2)	2602 (35.3)	532 (47.5)	409 (51.8)	79 (41.6)	56 (30.3)	147 (53.5)
*Unknown*	3893 (5.7)	676 (9.2)	84 (7.5)	117 (14.8)	31 (16.3)	18 (9.7)	21 (7.6)
CCI before dementia diagnosis, median (IQR)	1.0 (2.0)	1.0 (3.0)	1.0 (3.0)	1.0 (3.0)	1.0 (2.0)	1.0 (2.0)	1.0 (2.0)
Comorbidities before dementia diagnosis, n (%)							
*Atrial fibrillation*	12,380 (18.1)	1231 (16.7)	192 (17.2)	79 (10.0)	17 (8.9)	30 (16.2)	18 (6.5)
*Cancer*	15,539 (22.7)	1451 (19.7)	177 (15.8)	92 (11.6)	13 (6.8)	54 (29.2)	32 (11.6)
*Cerebrovascular diseases*	13,779 (20.2)	1484 (20.1)	234 (20.9)	124 (15.7)	39 (20.5)	44 (23.8)	47 (17.1)
*Congestive heart failure*	7795 (11.4)	944 (12.8)	174 (15.5)	95 (12.0)	19 (10.0)	20 (10.8)	22 (8.0)
*Chronic obstructive pulmonary diseases*	3592 (5.3)	526 (7.1)	76 (6.8)	35 (4.4)	6 (3.2)	10 (5.4)	11 (4.0)
*Diabetes*	3629 (5.3)	450 (6.1)	117 (10.5)	128 (16.2)	24 (12.6)	7 (3.8)	30 (10.9)
*Hypertensive diseases*	30,362 (44.4)	3525 (47.8)	527 (47.1)	325 (41.1)	77 (40.5)	72 (38.9)	102 (37.1)
*Liver diseases*	451 (0.7)	77 (1.0)	13 (1.2)	14 (1.8)	6 (3.2)	2 (1.1)	5 (1.8)
*Myocardial infarction*	7750 (11.3)	936 (12.7)	160 (14.3)	118 (14.9)	7 (3.7)	23 (12.4)	16 (5.8)
*Peripheral vascular diseases*	3728 (5.5)	452 (6.1)	60 (5.4)	32 (4.1)	7 (3.7)	8 (4.3)	11 (4.0)
*Renal diseases*	2232 (3.3)	230 (3.1)	44 (3.9)	42 (5.3)	11 (5.8)	2 (1.1)	10 (3.6)
*Rheumatic diseases*	4800 (7.0)	471 (6.4)	55 (4.9)	47 (5.9)	10 (5.3)	8 (4.3)	14 (5.1)
Drug use during one year before dementia diagnosis, n (%)							
*ACEi/ARBs*	26,993 (39.5)	3170 (43.0)	486 (43.4)	349 (44.2)	66 (34.7)	65 (35.1)	109 (39.6)
*Antidepressants*	21,786 (31.9)	2178 (29.6)	409 (36.6)	277 (35.1)	56 (29.5)	55 (29.7)	103 (37.5)
*Antipsychotics*	4161 (6.1)	547 (7.4)	104 (9.3)	55 (7.0)	20 (10.5)	11 (5.9)	18 (6.5)
*Anxiolytics*	11,798 (17.3)	1199 (16.3)	238 (21.3)	155 (19.6)	24 (12.6)	25 (13.5)	43 (15.6)
*Beta blockers*	26,308 (38.5)	2960 (40.2)	448 (40.0)	301 (38.1)	48 (25.3)	60 (32.4)	75 (27.3)
*Calcium channel blockers*	16,269 (23.8)	2063 (28.0)	291 (26.0)	180 (22.8)	50 (26.3)	42 (22.7)	62 (22.5)
*Diuretics*	20,176 (29.5)	2209 (30.0)	371 (33.2)	200 (25.3)	37 (19.5)	43 (23.2)	62 (22.5)
*Hypnotics*	17,092 (25.0)	1994 (27.1)	277 (24.8)	208 (26.3)	41 (21.6)	51 (27.6)	71 (25.8)
*Statins*	22,980 (33.6)	2612 (35.5)	446 (39.9)	339 (42.9)	55 (28.9)	61 (33.0)	82 (29.8)

IQR, Inter Quartile Range; CCI, Charlson Comorbidity Index; ACEi/ARBs, Angiotensin-converting enzyme inhibitors/Angiotensin II receptor blockers.

### 3.2 Description of dementia diagnostics and drug prescription

Table 2 illustrates the dementia diagnostics and the prescription of anti-dementia drugs among foreign-born and Swedish-born persons with dementia. Swedish-born individuals had the highest proportion of receiving a dementia diagnosis in primary care (n = 31,102, 45.5%), compared to the other groups. Meanwhile, dementia diagnosis in the memory clinics was more common among people born in Asia (76.7%), Africa (81.6%) and South America (85.8%). There was a statistically significant difference in the basic diagnostic work-up in relation to regions of birth. South American-born persons with dementia had the highest proportion of completing basic diagnostic work-up (n = 209, 79.8%), while the other European countries, Asian- and African-born people had the lowest proportions. Similar trends occurred in clock test and MMSE, where the other European countries, Asia and Africa groups had the lowest percentages of completion. Swedish-born persons with dementia had the lowest proportion of receiving CT-MRI (n = 59,422, 89.3%), compared to foreign-born people. People born in Africa and the other European countries had the lowest proportions of receiving cholinesterase inhibitors (64.5% and 66.0%, respectively), however, the highest proportions of receiving memantine (51.3% and 52.5%, respectively).

**Table 2:** Dementia diagnostics and anti-dementia drug prescription of dementia patients from different regions of birth (n = 78,252).

	Sweden (n = 68,326)	Other EU & Nordic countries (n = 7367)	The other European countries (n = 1119)	Asia (n = 790)	Africa (n = 190)	North America (n = 185)	South America (n = 275)
Year of dementia diagnosis, n (%)							
*2007–2010*	14,565 (21.3)	1510 (20.5)	210 (18.8)	111 (14.1)	25 (13.2)	45 (24.3)	61 (22.2)
*2011–2018*	53,761 (78.7)	5857 (79.5)	909 (81.2)	679 (85.9)	165 (86.8)	140 (75.7)	214 (77.8)
Types of diagnostic unit, n (%)							
*Primary care*	31,102 (45.5)	2909 (39.5)	430 (38.4)	184 (23.3)	35 (18.4)	63 (34.1)	39 (14.2)
*Memory clinic*	37,224 (54.5)	4458 (60.5)	689 (61.6)	606 (76.7)	155 (81.6)	122 (65.9)	236 (85.8)
Dementia types, n (%)							
*Alzheimer's disease*	21,525 (31.5)	2220 (30.1)	290 (25.9)	198 (25.1)	41 (21.6)	58 (31.4)	94 (34.2)
*Mixed dementia*	12,644 (18.5)	1600 (21.7)	213 (19.0)	161 (20.4)	27 (14.2)	30 (16.2)	70 (25.5)
*Vascular dementia*	12,928 (18.9)	1437 (19.5)	245 (21.9)	192 (24.3)	55 (28.9)	45 (24.3)	45 (16.4)
*Lewy body dementia*	1501 (2.2)	119 (1.6)	18 (1.6)	20 (2.5)	4 (2.1)	3 (1.6)	4 (1.5)
*Frontotemporal dementia*	1058 (1.5)	85 (1.2)	21 (1.9)	16 (2.0)	6 (3.2)	4 (2.2)	3 (1.1)
*Parkinson disease with dementia*	1045 (1.5)	89 (1.2)	14 (1.3)	6 (0.8)	4 (2.1)	2 (1.1)	5 (1.8)
*Unspecified dementia*	15,867 (23.2)	1603 (21.8)	282 (25.2)	174 (22.0)	43 (22.6)	38 (20.5)	49 (17.8)
*Other dementias*	1758 (2.6)	214 (2.9)	36 (3.2)	23 (2.9)	10 (5.3)	5 (2.7)	5 (1.8)
Dementia diagnostic examination, n (%)							
*Basic diagnostic work-up*^a^	50,247 (77.4)	5467 (77.7)	662 (63.0)	489 (65.1)	115 (63.2)	137 (78.3)	209 (79.8)
*Clock test*	58,656 (88.3)	6219 (86.9)	818 (75.9)	570 (75.1)	132 (71.0)	150 (84.3)	234 (87.6)
*Blood analysis*	63,535 (95.5)	6873 (95.3)	1004 (93.0)	739 (95.8)	180 (96.3)	172 (96.1)	256 (95.9)
*Mini-Mental State Examination*	64,308 (94.1)	6832 (92.7)	884 (79.0)	614 (77.7)	156 (82.1)	169 (91.4)	248 (90.2)
*MMSE score, median (IQR)*	22.0 (7.0)	20.0 (7.0)	17.0 (8.0)	18.0 (9.0)	16.0 (11.0)	22.0 (6.0)	19.0 (7.0)
*CT-MRI*	59,422 (89.3)	6640 (92.2)	976 (90.5)	733 (95.1)	177 (94.7)	168 (92.3)	252 (94.0)
*Neuropsychological assessment*	11,414 (17.5)	1276 (18.0)	106 (10.1)	123 (16.2)	31 (16.7)	40 (23.1)	52 (19.6)
*Occupational therapy assessment*	27,661 (42.1)	3424 (48.1)	446 (41.8)	374 (49.1)	97 (52.7)	94 (52.8)	157 (59.0)
*Lumbar puncture*	17,421 (26.5)	1995 (28.0)	275 (26.0)	279 (36.6)	90 (48.6)	49 (27.7)	115 (42.9)
Anti-dementia drugs b, n (%)							
*Cholinesterase inhibitors*	26,943 (73.4)	2792 (69.3)	353 (66.0)	287 (74.5)	49 (64.5)	64 (68.8)	128 (74.0)
*Memantine*	16,888 (46.0)	1854 (46.0)	281 (52.5)	162 (42.1)	39 (51.3)	39 (41.9)	79 (45.7)

MMSE, Mini-Mental State Examination; CT, computed tomography; MRI, magnetic resonance imaging.

a The basic diagnostic work-up meant whether patients received all basic tests (clock test, blood analysis, MMSE, CT-MRI) or not.

b Only persons with Alzheimer's disease, mixed dementia, Lewy Body dementia or Parkinson's disease dementia (n = 42, 005) were analyzed for Cholinesterase inhibitors and Memantine (Sweden (n = 36,715), The other EU & Nordic countries (n = 4028), The other European countries (n = 535), Asia (n = 385), Africa (n = 76), North America (n = 93), South America (n = 173)).

### 3.3 Dementia diagnostics and drug prescription in relation to region of birth

As shown in Table 3, region of birth was significantly associated with the completion of basic diagnostic work-up. Compared to Swedish-born persons with dementia, lower odds of receiving basic diagnostic work-up were seen in individuals born in Africa (OR 0.31, 95% CI 0.22–0.43), Asia (OR 0.45, 95% CI 0.38–0.53), and the other European countries (OR 0.50, 95% CI 0.43–0.57). Similarly, the other Nordic and European countries, Asia- and African-born individuals were less likely to receive the clock test, MMSE and neuropsychological assessment. Regarding anti-dementia drugs, people who were born in the other Nordic and European countries or Africa had lower chances of receiving cholinesterase inhibitors, compared to Swedish-born individuals. Asian-born people were less likely to get memantine, compared to native people (OR 0.61, 95% CI 0.49–0.76).

**Table 3:** Region of birth in association with dementia diagnostics and the use of anti-dementia drugs (n = 78,252).

		Model 1	Model 2	Model 3
Basic diagnostic work-up	Sweden	reference	reference	reference
	Other EU & Nordic countries	1.02 (0.96, 1.08)	0.98 (0.92, 1.04)	0.99 (0.93, 1.05)
	The other European countries	0.50 (0.44, 0.56)	0.42 (0.37, 0.48)	0.50 (0.43, 0.57)
	Asia	0.54 (0.47, 0.63)	0.41 (0.35, 0.48)	0.45 (0.38, 0.53)
	Africa	0.50 (0.37, 0.68)	0.32 (0.23, 0.43)	0.31 (0.22, 0.43)
	North America	1.05 (0.73, 1.50)	1.13 (0.79, 1.63)	1.05 (0.72, 1.53)
	South America	1.15 (0.85, 1.55)	0.98 (0.72, 1.33)	0.92 (0.67, 1.27)
	p-value of Wald test	<0.001	<0.001	<0.001
	p-value of Hosmer-Lemeshow test	Not applicable	<0.001	<0.001
Clock test	Sweden	reference	reference	reference
	Other EU & Nordic countries	0.88 (0.82, 0.95)	0.85 (0.79, 0.92)	0.91 (0.85, 0.99)
	The other European countries	0.42 (0.36, 0.48)	0.38 (0.33, 0.43)	0.51 (0.43, 0.59)
	Asia	0.40 (0.34, 0.47)	0.33 (0.28, 0.39)	0.46 (0.38, 0.55)
	Africa	0.33 (0.24, 0.45)	0.24 (0.17, 0.33)	0.29 (0.21, 0.41)
	North America	0.71 (0.48, 1.07)	0.75 (0.50, 1.12)	0.73 (0.48, 1.10)
	South America	0.94 (0.65, 1.36)	0.84 (0.58, 1.21)	1.04 (0.70, 1.53)
	p-value of Wald test	<0.001	<0.001	<0.001
	p-value of Hosmer-Lemeshow test	Not applicable	<0.001	<0.001
Blood analysis	Sweden	reference	reference	reference
	Other EU & Nordic countries	0.95 (0.85, 1.07)	0.93 (0.83, 1.04)	0.97 (0.86, 1.09)
	The other European countries	0.62 (0.49, 0.79)	0.58 (0.46, 0.74)	0.69 (0.54, 0.89)
	Asia	1.08 (0.75, 1.54)	0.95 (0.67, 1.36)	1.05 (0.72, 1.53)
	Africa	1.20 (0.56, 2.55)	0.99 (0.46, 2.10)	1.05 (0.49, 2.26)
	North America	1.15 (0.54, 2.44)	1.19 (0.56, 2.54)	1.11 (0.52, 2.38)
	South America	1.09 (0.59, 1.99)	0.99 (0.54, 1.82)	0.99 (0.54, 1.82)
	p-value of Wald test	0.013	0.002	0.185
	p-value of Hosmer-Lemeshow test	Not applicable	<0.001	<0.001
MMSE	Sweden	reference	reference	reference
	Other EU & Nordic countries	0.80 (0.73, 0.88)	0.78 (0.71, 0.86)	0.83 (0.75, 0.91)
	The other European countries	0.24 (0.20, 0.27)	0.22 (0.19, 0.25)	0.31 (0.26, 0.37)
	Asia	0.22 (0.18, 0.26)	0.19 (0.16, 0.23)	0.27 (0.22, 0.34)
	Africa	0.29 (0.20, 0.42)	0.23 (0.16, 0.34)	0.29 (0.20, 0.43)
	North America	0.66 (0.39, 1.10)	0.68 (0.41, 1.14)	0.67 (0.40, 1.13)
	South America	0.57 (0.39, 0.85)	0.53 (0.36, 0.79)	0.66 (0.43, 1.02)
	p-value of Wald test	<0.001	<0.001	<0.001
	p-value of Hosmer-Lemeshow test	Not applicable	<0.001	<0.001
CT-MRI	Sweden	reference	reference	reference
	Other EU & Nordic countries	1.41 (1.29, 1.54)	1.32 (1.20, 1.44)	1.21 (1.10, 1.33)
	The other European countries	1.14 (0.93, 1.39)	0.90 (0.73, 1.10)	0.91 (0.72, 1.15)
	Asia	2.31 (1.67, 3.21)	1.57 (1.13, 2.19)	1.13 (0.79, 1.61)
	Africa	2.12 (1.12,4.02)	1.11 (0.58, 2.13)	0.75 (0.38, 1.50)
	North America	1.44 (0.83, 2.48)	1.63 (0.94, 2.83)	1.46 (0.81, 2.65)
	South America	1.89 (1.14, 3.13)	1.53 (0.92, 2.56)	0.89 (0.51, 1.55)
	p-value of Wald test	<0.001	<0.001	0.005
	p-value of Hosmer-Lemeshow test	Not applicable	<0.001	<0.001
Neuropsychological assessment	Sweden	reference	reference	reference
	Other EU & Nordic countries	1.04 (0.97, 1.11)	0.99 (0.92, 1.05)	0.85 (0.79, 0.92)
	The other European countries	0.53 (0.43, 0.65)	0.40 (0.32, 0.49)	0.33 (0.26, 0.41)
	Asia	0.91 (0.75, 1.11)	0.56 (0.46, 0.69)	0.41 (0.33, 0.51)
	Africa	0.94 (0.64, 1.39)	0.43 (0.29, 0.64)	0.27 (0.18, 0.41)
	North America	1.42 (1.00, 2.02)	1.62 (1.12, 2.35)	1.03 (0.69, 1.54)
	South America	1.15 (0.85, 1.56)	0.86 (0.62, 1.18)	0.54 (0.39, 0.75)
	p-value of Wald test	<0.001	<0.001	<0.001
	p-value of Hosmer-Lemeshow test	Not applicable	0.041	<0.001
Occupational therapy assessment	Sweden	reference	reference	reference
	Other EU & Nordic countries	1.28 (1.21, 1.34)	1.27 (1.21, 1.34)	1.15 (1.10, 1.22)
	The other European countries	0.99 (0.88, 1.12)	0.99 (0.88, 1.12)	0.91 (0.80, 1.04)
	Asia	1.33 (1.15, 1.53)	1.34 (1.16, 1.54)	1.09 (0.93, 1.27)
	Africa	1.53 (1.15, 2.05)	1.56 (1.16, 2.08)	1.19 (0.88, 1.61)
	North America	1.54 (1.15, 2.07)	1.54 (1.15, 2.07)	1.35 (1.00, 1.84)
	South America	1.98 (1.55, 2.53)	1.98 (1.55, 2.53)	1.55 (1.20, 2.00)
	p-value of Wald test	<0.001	<0.001	<0.001
	p-value of Hosmer-Lemeshow test	Not applicable	0.120	<0.001
Lumbar puncture	Sweden	reference	reference	reference
	Other EU & Nordic countries	1.08 (1.02, 1.14)	1.00 (0.94, 1.06)	0.96 (0.90, 1.03)
	The other European countries	0.98 (0.85, 1.12)	0.69 (0.59, 0.80)	0.71 (0.59, 0.84)
	Asia	1.60 (1.38, 1.86)	0.89 (0.76, 1.06)	0.79 (0.65, 0.95)
	Africa	2.63 (1.97, 3.52)	1.11 (0.80, 1.55)	0.84 (0.59, 1.21)
	North America	1.06 (0.77, 1.48)	1.33 (0.93, 1.92)	0.97 (0.65, 1.44)
	South America	2.09 (1.64, 2.66)	1.55 (1.18, 2.04)	1.07 (0.80, 1.42)
	p-value of Wald test	<0.001	<0.001	0.001
	p-value of Hosmer-Lemeshow test	Not applicable	<0.001	<0.001
Cholinesterase inhibitors	Sweden	reference	reference	reference
	Other EU & Nordic countries	0.82 (0.76, 0.88)	0.75 (0.69, 0.81)	0.87 (0.80, 0.94)
	The other European countries	0.70 (0.59, 0.84)	0.59 (0.48, 0.71)	0.73 (0.60, 0.90)
	Asia	1.06 (0.84, 1.34)	0.72 (0.56, 0.91)	1.00 (0.77, 1.30)
	Africa	0.66 (0.41, 1.05)	0.36 (0.21, 0.59)	0.44 (0.26, 0.75)
	North America	0.80 (0.52, 1.24)	0.90 (0.57, 1.43)	0.88 (0.55, 1.41)
	South America	1.03 (0.73, 1.45)	0.78 (0.55, 1.12)	1.13 (0.78, 1.64)
	p-value of Wald test	<0.001	<0.001	<0.001
	p-value of Hosmer-Lemeshow test	Not applicable	<0.001	<0.001
Memantine	Sweden	reference	reference	reference
	Other EU & Nordic countries	1.00 (0.94, 1.07)	0.98 (0.92, 1.05)	0.97 (0.91, 1.04)
	The other European countries	1.30 (1.09, 1.54)	1.21 (1.02, 1.44)	1.15 (0.96, 1.38)
	Asia	0.85 (0.70, 1.05)	0.68 (0.55, 0.83)	0.61 (0.49, 0.76)
	Africa	1.24 (0.79, 1.94)	0.94 (0.59, 1.49)	0.83 (0.52, 1.33)
	North America	0.85 (0.56, 1.28)	0.88 (0.58, 1.34)	0.85 (0.55, 1.30)
	South America	0.99 (0.73, 1.33)	0.86 (0.63, 1.17)	0.75 (0.55, 1.02)
	p-value of Wald test	0.044	0.003	<0.001
	p-value of Hosmer-Lemeshow test	Not applicable	<0.001	0.120

Results from binary logistic regression, presented as odds ratios (95% confidence interval).

Model 1 was unadjusted.

Model 2 was adjusted for age at dementia diagnosis and sex.

Model 3 was additionally adjusted for living alone, living areas (urban/intermediate/rural), education, income, Charlson Comorbidity Index, year of diagnosis and type of diagnosis unit.

Basic dementia diagnostic work-up refers to the completion of four tests: clock test, blood analysis, Mini-Mental State Examination (MMSE) and computed tomography or magnetic resonance imaging (CT-MRI).

Only persons with Alzheimer's disease, mixed dementia, Lewy Body dementia or Parkinson's disease dementia (n = 42,005) were analyzed for Cholinesterase inhibitors and Memantine.

### 3.4 Sensitivity analyses

Compared to Swedish-born individuals, stratified analysis showed that both men and women born in the other European countries, Asia and Africa were less likely to receive the basic diagnostic work-up, clock test, MMSE and neuropsychological assessment (Supplementary Table 4). Subgroup analysis on the type of diagnostic unit showed lower likelihood of getting clock test and MMSE when people born in the other European countries, Asia and Africa were diagnosed in either primary care or memory clinics (Supplementary Table 5). When additionally adjusting for MMSE score, several of the above associations were not statistically significant (Supplementary Table 7). African-born people had a lower chance of receiving the clock test (OR 0.62, 95% CI 0.43–0.90). Asian-born people were more likely to receive cholinesterase inhibitors (OR 1.44, 95% CI 1.10–1.88), but less likely to get memantine (OR 0.56, 95% CI 0.45–0.69), compared to Swedish-born persons.

## 4 Discussion

Our study explored the disparities between Swedish-born and foreign-born individuals in dementia diagnostics and the use of anti-dementia drugs. The main findings of this study are that region of birth was significantly associated with performance of basic dementia diagnostic work-up, individual diagnostic tests, and the prescription of anti-dementia drugs. People born in the other European countries, Asia and Africa were less likely to receive the basic diagnostic work-up, clock test, MMSE and neuropsychological assessment. Lower chances of receiving anti-dementia drugs were observed in people born in the other Nordic and European countries or Africa (cholinesterase inhibitors), and Asia (memantine). Similar results were seen in the subgroup analyses on sex, type of diagnostic unit and year of diagnosis. In sensitivity analysis controlled for MMSE, some of the above associations became non-significant.

Our results concur with previous studies in the Nordic countries, such as Denmark (Nielsen et al., 2011; Stevnsborg et al., 2016), or Norway (Diaz et al., 2015), and other high-income countries, such as the USA (Mehta et al., 2005; Poon et al., 2009). A recent study in Sweden also showed that the foreign-born people were less likely to receive dementia diagnosis and cholinesterase inhibitors, compared to the Swedish-born persons (Lindgren et al., 2021). Compared to Lindgren et al., 2021, in our study, we further categorized the country of birth according to geographical locations, which denoted the origin of persons with dementia more precisely than classifying by national economy. Moreover, we combined data from SveDem with various nationwide registries on diseases, drug prescription and sociodemographic to adjust for various kinds of confounding factors for dementia diagnostics and treatment (meanwhile, the above study focused on information retrieved from SveDem). In addition, we considered people diagnosed with early-onset dementia (less than 65 years old at dementia diagnosis).

The lower access to dementia diagnostics and anti-dementia drugs might be explained by patient-related factors, such as culture, language proficiency, health literacy, and socioeconomic status, or due to institutional factors in the health-care system. Foreign-born individuals with poor acculturation or limited language proficiency possibly have trouble in reaching appropriate healthcare services (Ngo-Metzger et al., 2003), although trained medical interpreters or bilingual providers are available. Furthermore, the validity and the reliability of testing could be questioned if performed with the assistance of an interpreter. Interpreters are readily available in Sweden and provided free for all healthcare encounters. In our study, persons with dementia born in Asia, Africa and the other European countries had lower likelihood of receiving clock test and MMSE. Low proficiency in Swedish or English might be a barrier for these tests. In line with this, the lack of difference regarding blood analysis or CT-MRI can be explained because these tests do not require language proficiency in Swedish. In our study, MMSE scores at the time of diagnosis among persons with dementia born in Asia, Africa and the other European countries were significantly lower, compared to those among Swedish-born people. This may mean that they were diagnosed later compared to the natives, or due to confounding from different educational attainment or language.

The lack of knowledge of local healthcare, social services and transportation has been identified as an obstacle leading to lower access to healthcare services among foreign-born people (Daker-White, Beattie, Gilliard, & Means, 2002; van Wezel et al., 2016). Foreign-born persons have been shown to visit the hospital less frequently compared to native citizens (Ku & Matani, 2001). For some immigrant groups, there may be cultural expectations of receiving care within the family, or unawareness of support groups and healthcare resources available to them (Kajiyama et al., 2013; Lee, Ralston, Drey, Partridge, & Rosen, 2005; Mukadam, Cooper, Basit, & Livingston, 2011). A recent study has shown that immigrants hesitate to seek formal care and do so later than native Swedes (Rosendahl, Söderman, & Mazaheri, 2016).

Furthermore, socioeconomic status is an important factor that possibly affects the dementia diagnostics and drug prescription (Hoang et al., 2021; Johnell, Weitoft, & Fastbom, 2008). Our study showed persons with dementia born in Asia, Africa, South America, and the other European countries were overrepresented in the lowest education or income categories. The ‘unknown’ education accounted for the largest part in Asian and African groups, which could reflect lower educational levels. Moreover, previous studies have also pointed out the impact of socioeconomic status on dementia diagnostics and drug prescription, in which people with lower education or income were less likely to receive dementia diagnostics and drug prescription, compared to ones with higher education or income (Hoang et al., 2021; Johnell et al., 2008).

A unique feature of our study is that we considered the influence of cognitive impairment of persons with dementia (partly shown by MMSE scores) on the association between region of birth and dementia diagnostics and the prescription of anti-dementia drugs, while previous studies did not. MMSE is a reliable and valid tool for examining cognitive impairment (Folstein et al., 1975; Pezzotti et al., 2008). However, MMSE performance was related to educational attainment and hindered by language barriers or culture (Crum et al., 1993; Nielsen & Jørgensen, 2020). Controlling for MMSE score is still necessary when assessing these kinds of health inequalities, because the types of diagnostic tests and drugs that clinicians prescribe may depend on the degree of cognitive impairment. In our study, after adjusting for MMSE, several associations between region of birth and dementia diagnostics or the use of anti-dementia drugs were not significant (Supplementary Table 7). Thus, the disparities in dementia care between Swedish-born and foreign-born people might be explained by the fact that foreign-born people had more severe dementia at the time of diagnosis, reflected in their lower MMSE scores (Table 2). In addition, a preceding study showed that high age and degree of cognitive impairment influenced the choice of diagnostic tests (Religa et al., 2012).

### 4.1 Limitations

There were several limitations in our study. First, region of birth cannot totally describe immigration status or ethnicity. Ethnicity is not registered in Sweden, by law. Individual country of origin and classification of countries based on income were not available in this study. Data on specific country of birth are available in LISA, but rarely delivered to medical researchers in Sweden due to privacy concerns (Ludvigsson et al., 2019). Moreover, this is an observational study and does not attempt to assess causality. Thus, we acknowledge the possibility of residual confounding.

The main strength of this study is the linkage of national quality registers, which enables a large sample size, supports the generalizability of the study, and reduces non-participation bias. Another advantage is a large sample of persons with dementia from SveDem, which is the largest clinical dementia registry in the world.

## 5 Conclusions

To conclude, health inequalities in dementia diagnostics and the prescription of anti-dementia drugs existed in this cohort. Region of birth was associated with performance of basic dementia diagnostic work-up, individual diagnostic tests, and the use of anti-dementia drugs. Being born in the other European countries, Asia and Africa was associated with lower likelihood of receiving the basic diagnostic work-up, clock test, MMSE and neuropsychological assessment, compared to being born in Sweden. Lower chances of receiving anti-dementia drugs were seen in people born in the other Nordic and European countries or Africa (cholinesterase inhibitors), and Asia (memantine). However, some of these associations were not significant when additionally adjusting for MMSE scores. Government-funded universal healthcare coverage cannot completely solve the problem of health inequality. Whether discrepancies in dementia care were due to the immigration status and ethnicity or because of the more vulnerable health status of immigrants should be considered and investigated in future studies.

## Brief summary

Disparity in dementia diagnostics and drug prescription was not obvious among immigrants in Sweden. Immigration was associated with the receipt of basic dementia diagnostic work-up, individual diagnostic tests, type of diagnostic unit and anti-dementia drugs, but most of these associations were not significant after adjusting for MMSE scores.

## Funding sources

This study was supported by FORTE - the Swedish Council for Health, Working Life and Welfare (Grant 2017–01646), Johanniterorden I Sverige/Swedish Order of St. John, Stiftelsen för Sigurd och Elsa Goljes Minne, Svenska Sällskapet för Medicinsk Forskning, StratNeuro and NHMRC-ARC Dementia Research Development Fellowship (Grant 1107381).

## Ethical considerations

Ethical approval was granted by the Swedish Ethical Review Authority (decision number 2017/501-31; 2017/1448-32; 2021-05289). All persons with dementia were informed about the registration in the registers and their data might be used for quality improvement or research purposes. They could refuse to participate or withdraw consent at any time. Their identities were anonymized and blinded to the researchers.

## Authors' contributions

• Study concept and design: Minh Tuan Hoang, Ingemar Kåreholt, Sara Garcia-Ptacek.

• Acquisition of data: Minh Tuan Hoang, Ingemar Kåreholt, Sara Garcia-Ptacek, Hong Xu.

• Registers preparation: Hong Xu, Edwin C.K Tan, Kristina Johnell.

• Analysis and interpretation of data: Minh Tuan Hoang, Ingemar Kåreholt, Emma Lindgren.

• Clinical perspectives: Lena von Koch, Katarina Nägga, Maria Eriksdotter, Sara Garcia-Ptacek.

• Drafting of the manuscript: Minh Tuan Hoang.

• Critical revision of the manuscript for important intellectual content: all authors.

• Final approval of the version to be published: all authors.

## Declaration of competing interest

The authors declare that they have no known competing financial interests or personal relationships that could have appeared to influence the work reported in this paper.

## Data Availability

The authors do not have permission to share data.

## Appendix A Supplementary data

Multimedia component 1 Multimedia component1
