# Supplementary material for: Immigration and access to dementia diagnostics and treatment: A nationwide study in Sweden
Source: SSM Popul Health. 2023 Dec 2;25:101573. doi: 10.1016/j.ssmph.2023.101573 (PMC10755485; doi:10.1016/j.ssmph.2023.101573)
Supplement: Multimedia component 1 [file mmc1.docx]

**Immigration and access to dementia diagnostics and treatment:**

**A nationwide study in Sweden**

**SUPPLEMENTARY MATERIALS**

**Supplementary materials**

[Supplementary Table 1. The RECORD statement – checklist of items, extended from the STROBE statement, that should be reported in observational studies using routinely collected health data 1](#_Toc133451272)

[Supplementary Table 2. ICD-10 Codes for comorbidities 3](#_Toc133451273)

[Supplementary Table 3. ATC Codes for drug prescription 4](#_Toc133451274)

[Supplementary Table 4. Region of birth in association with dementia diagnostics and the use of anti-dementia drugs, stratified by sex 5](#_Toc133451275)

[Supplementary Table 5. Region of birth in association with dementia diagnostics and the use of anti-dementia drugs, stratified by types of diagnostic unit 7](#_Toc133451276)

[Supplementary Table 6. Region of birth in association with dementia diagnostics and the use of anti-dementia drugs, stratified by year of diagnosis 9](#_Toc133451277)

[Supplementary Table 7. Region of birth in association with dementia diagnostics and the use of anti-dementia drugs, additionally adjusted by MMSE score at baseline 11](#_Toc133451278)

[References 13](#_Toc133451279)

##

## Supplementary Table 1. The RECORD statement – checklist of items, extended from the STROBE statement, that should be reported in observational studies using routinely collected health data (1)

| **RECORD items** | | **Details** | **Location** |
| --- | --- | --- | --- |
| **Title and abstract** | 1 | (1) The type of data used should be specified in the title or abstract. When possible, the name of the databases used should be included. | Abstract |
|  |  | (2) If applicable, the geographic region and timeframe within which the study took place should be reported in the title or abstract. | Abstract |
|  |  | (3) If linkage between databases was conducted for the study, this should be clearly stated in the title or abstract. | Abstract |
| **Introduction** | | |  |
| Background/rationale | 2 | Explain the scientific background and rationale for the investigation being reported | Introduction > 1^st^ and 2^nd^ paragraphs |
| Objectives | 3 | State specific objectives, including any prespecified hypotheses | Introduction > Last paragraph |
| **Methods** | | |  |
| Study design | 4 | Present key elements of study design early in the paper | Methods > Study design and setting |
| Setting | 5 | Describe the setting, locations, and relevant dates, including periods of recruitment, exposure, follow-up, and data collection | Methods > Study design and setting |
| Participants | 6 | (*1*) The methods of study population selection (such as codes or algorithms used to identify subjects) should be listed in detail. If this is not possible, an explanation should be provided. | Methods > Participants |
|  |  | (*2*) Any validation studies of the codes or algorithms used to select the population should be referenced. If validation was conducted for this study and not published elsewhere, detailed methods and results should be provided. | Not applicable |
|  |  | (3) If the study involved linkage of databases, consider use of a flow diagram or other graphical display to demonstrate the data linkage process, including the number of individuals with linked data at each stage. | Figure 1 |
| Variables | 7 | A complete list of codes and algorithms used to classify exposures, outcomes, confounders, and effect modifiers should be provided. If these cannot be reported, an explanation should be provided. | Methods > Variables & Data sources |
| Data sources/ measurement | 8* | For each variable of interest, give sources of data and details of methods of assessment (measurement). Describe comparability of assessment methods if there is more than one group | Methods > Variables & Data sources |
| Bias | 9 | Describe any efforts to address potential sources of bias | Discussion > Limitations |
| Study size | 10 | Explain how the study size was arrived at | Methods > Participants & Figure 1 |
| Quantitative variables | 11 | Explain how quantitative variables were handled in the analyses. If applicable, describe which groupings were chosen and why | Methods > Variables & Data sources |
| Statistical methods | 12 | (*a*) Describe all statistical methods, including those used to control for confounding | Methods > Statistical analysis |
|  |  | (*b*) Describe any methods used to examine subgroups and interactions | Methods > Statistical analysis |
|  |  | (*c*) Explain how missing data were addressed | Methods > Statistical analysis |
|  |  | (*d*) If applicable, explain how loss to follow-up was addressed | Not applicable |
|  |  | (*e*) Describe any sensitivity analyses | Methods > Statistical analysis |
| Data access and cleaning methods |  | Authors should describe the extent to which the investigators had access to the database population used to create the study population. | Methods > Study design and data sources |
|  |  | Authors should provide information on the data cleaning methods used in the study. | Not applicable |
|  |  | State whether the study included person-level, institutional-level, or other data linkage across two or more databases. The methods of linkage and methods of linkage quality evaluation should be provided. | Methods > Study design and setting |
| **Results** | | |  |
| Participants | 13* | (a) Describe in detail the selection of the persons included in the study (i.e., study population selection) including filtering based on data quality, data availability and linkage. The selection of included persons can be described in the text and/or by means of the study flow diagram. | Methods > Participants & Figure 1 |
|  |  | (b) Give reasons for non-participation at each stage | Methods > Participants & Figure 1 |
|  |  | (c) Consider use of a flow diagram | Methods > Participants & Figure 1 |
| Descriptive data | 14* | (a) Give characteristics of study participants (eg demographic, clinical, social) and information on exposures and potential confounders | Results > Description of the Study Population & Table 1 |
|  |  | (b) Indicate number of participants with missing data for each variable of interest | Methods > Participants & Figure 1 |
|  |  | (c) Summarise follow-up time (eg, average and total amount) | Methods > Participants & Figure 1 |
| Outcome data | 15* | Report numbers of outcome events or summary measures over time | Results & Table 2 |
| Main results | 16 | (*a*) Give unadjusted estimates and, if applicable, confounder-adjusted estimates and their precision (eg, 95% confidence interval). Make clear which confounders were adjusted for and why they were included | Results & Table 3 |
|  |  | (*b*) Report category boundaries when continuous variables were categorized | Not applicable |
|  |  | (*c*) If relevant, consider translating estimates of relative risk into absolute risk for a meaningful time period | Not applicable |
| Other analyses | 17 | Report other analyses done—eg analyses of subgroups and interactions, and sensitivity analyses | Supplementary Tables 4, 5, 6, 7 |
| **Discussion** | | |  |
| Key results | 18 | Summarise key results with reference to study objectives | Discussion > First paragraph |
| Limitations | 19 | Discuss the implications of using data that were not created or collected to answer the specific research question(s). Include discussion of misclassification bias, unmeasured confounding, missing data, and changing eligibility over time, as they pertain to the study being reported. | Discussion > Limitations |
| Interpretation | 20 | Give a cautious overall interpretation of results considering objectives, limitations, multiplicity of analyses, results from similar studies, and other relevant evidence | Discussion |
| Generalisability | 21 | Discuss the generalisability (external validity) of the study results | Discussion |
| **Other information** | | |  |
| Funding | 22 | Give the source of funding and the role of the funders for the present study and, if applicable, for the original study on which the present article is based | Funding sources |
| Accessibility of protocol, raw data, and programming code |  | Authors should provide information on how to access any supplemental information such as the study protocol, raw data, or programming code. | Data availability statement |

## Supplementary Table 2. ICD-10 Codes for comorbidities *

| **Comorbidities** | **ICD-10 Codes (2)** |
| --- | --- |
| Atrial fibrillation | I48 |
| Cancer | C00 – C97 |
| Cerebrovascular diseases | G45,  I60, I61, I62, I63, I64, I67, I69 |
| Congestive heart failure | I110, I130, I132, I255, I420, I426, I427, I428, I429, I43, I50 |
| Chronic obstructive pulmonary disease | J43, J44 |
| Diabetes | E100 – E107, E110 – E117, E120 – E127, E130 -E137, E140 – E147 |
| Hypertensive diseases | I10, I11, I12, I13, I14, I15, I16 |
| Liver diseases | B15, B16, B17, B18, B19,  K754, K746, K73, K703, K709,  I850, I859, I982, I983 |
| Myocardial infarction | I21, I22, I252 |
| Peripheral vascular diseases | I70, I71, I731, I738, I739, I771, I790, I792,  K55 |
| Renal diseases | I120, I131,  Z992, Z940, Z49,  Q614, Q613, Q612, Q611,  N250, N19, N18, N11, N057, N056, N055, N054, N053, N052, N037, N036, N035, N034, N033, N032, Z992 |
| Rheumatic diseases | M05, M06, M070, M071, M072, M073, M08, M123, M13, M30, M313, M314, M315, M316, M32, M33, M34, M350, M351, M353, M45, M46 |
| * ICD, International Statistical Classification of Diseases and Related Health Problems. 10th revision. | |

## Supplementary Table 3. ATC Codes for drug prescription *

| **Drugs** | **ATC codes (3)** |
| --- | --- |
| ACEi/ARBs | C09 |
| Antidepressants | N06A |
| Antipsychotics | N05A |
| Anxiolytics | N05B |
| Beta blockers | C07 |
| Calcium channel blockers | C08 |
| Cholinesterase inhibitors | N06DA |
| Diuretics | C03 |
| Hypnotics | N05C |
| Memantine | N06DX01 |
| Statins | C10AA |
| * ATC, Anatomical Therapeutic Chemical Classification System | |

## Supplementary Table 4. Region of birth in association with dementia diagnostics and the use of anti-dementia drugs, stratified by sex

|  |  | **Female (n = 45,872)** | **Male (n = 32,380)** |
| --- | --- | --- | --- |
| Basic diagnostic work-up | Sweden | reference | reference |
|  | Other EU & Nordic countries | 0.98 (0.91, 1.06) | 0.99 (0.89, 1.09) |
|  | The other European countries | 0.47 (0.39, 0.57) | 0.53 (0.43, 0.66) |
|  | Asia | 0.32 (0.26, 0.41) | 0.66 (0.50, 0.86) |
|  | Africa | 0.19 (0.12, 0.30) | 0.52 (0.32, 0.87) |
|  | North America | 1.50 (0.84, 2.67) | 0.75 (0.45, 1.25) |
|  | South America | 0.90 (0.61, 1.32) | 0.96 (0.54, 1.69) |
|  | p-value | < 0.001 | < 0.001 |
| Clock test | Sweden | reference | reference |
|  | Other EU & Nordic countries | 0.87 (0.80, 0.96) | 0.98 (0.86, 1.12) |
|  | The other European countries | 0.44 (0.36, 0.54) | 0.61 (0.47, 0.78) |
|  | Asia | 0.34 (0.26, 0.43) | 0.69 (0.51, 0.93) |
|  | Africa | 0.21 (0.13, 0.34) | 0.44 (0.26, 0.76) |
|  | North America | 1.00 (0.52, 1.90) | 0.55 (0.32, 0.95) |
|  | South America | 1.26 (0.76, 2.08) | 0.72 (0.39, 1.31) |
|  | p-value | < 0.001 | < 0.001 |
| Blood analysis | Sweden | reference | reference |
|  | Other EU & Nordic countries | 1.03 (0.88, 1.20) | 0.88 (0.73, 1.06) |
|  | The other European countries | 0.70 (0.50, 0.98) | 0.67 (0.46, 0.99) |
|  | Asia | 0.94 (0.58, 1.54) | 1.15 (0.65, 2.06) |
|  | Africa | 0.77 (0.30, 1.95) | 1.73 (0.42, 7.09) |
|  | North America | 2.04 (0.50, 8.31) | 0.73 (0.29, 1.81) |
|  | South America | 0.72 (0.37, 1.37) | 3.73 (0.52, 26.85) |
|  | p-value | 0.350 | 0.175 |
| MMSE | Sweden | reference | reference |
|  | Other EU & Nordic countries | 0.82 (0.72, 0.92) | 0.85 (0.72, 0.99) |
|  | The other European countries | 0.29 (0.23, 0.36) | 0.33 (0.26, 0.43) |
|  | Asia | 0.21 (0.16, 0.27) | 0.38 (0.28, 0.52) |
|  | Africa | 0.18 (0.11, 0.30) | 0.63 (0.30, 1.34) |
|  | North America | 0.79 (0.36, 1.74) | 0.56 (0.28, 1.14) |
|  | South America | 0.70 (0.40, 1.21) | 0.58 (0.29, 1.19) |
|  | p-value | < 0.001 | < 0.001 |
| CT-MRI | Sweden | reference | reference |
|  | Other EU & Nordic countries | 1.22 (1.09, 1.37) | 1.18 (1.00, 1.39) |
|  | The other European countries | 1.02 (0.75, 1.38) | 0.76 (0.53, 1.09) |
|  | Asia | 1.02 (0.64, 1.62) | 1.29 (0.72, 2.30) |
|  | Africa | 0.47 (0.21, 1.02) | 3.22 (0.44, 23.81) |
|  | North America | 1.29 (0.60, 2.77) | 1.74 (0.68, 4.47) |
|  | South America | 0.72 (0.40, 1.30) | 3.03 (0.42, 22.12) |
|  | p-value | 0.001 | 0.098 |
| Neuropsychological assessment | Sweden | reference | reference |
|  | Other EU & Nordic countries | 0.80 (0.73, 0.88) | 0.94 (0.84, 1.05) |
|  | The other European countries | 0.31 (0.23, 0.43) | 0.35 (0.25, 0.47) |
|  | Asia | 0.33 (0.24, 0.46) | 0.49 (0.36, 0.65) |
|  | Africa | 0.26 (0.14, 0.49) | 0.28 (0.16, 0.50) |
|  | North America | 1.00 (0.55, 1.80) | 1.05 (0.60, 1.81) |
|  | South America | 0.51 (0.33, 0.78) | 0.59 (0.36, 0.97) |
|  | p-value | < 0.001 | < 0.001 |
| Occupational therapy assessment | Sweden | reference | reference |
|  | Other EU & Nordic countries | 1.24 (1.16, 1.32) | 1.03 (0.95, 1.12) |
|  | The other European countries | 0.90 (0.76, 1.08) | 0.95 (0.78, 1.15) |
|  | Asia | 1.17 (0.95, 1.45) | 1.03 (0.82, 1.29) |
|  | Africa | 1.11 (0.72, 1.72) | 1.30 (0.85, 1.99) |
|  | North America | 1.60 (1.03, 2.48) | 1.16 (0.75, 1.78) |
|  | South America | 1.47 (1.07, 2.02) | 1.76 (1.14, 2.72) |
|  | p-value | < 0.001 | 0.162 |
| Lumbar puncture | Sweden | reference | reference |
|  | Other EU & Nordic countries | 0.98 (0.90, 1.07) | 0.92 (0.83, 1.03) |
|  | The other European countries | 0.70 (0.55, 0.89) | 0.72 (0.56, 0.92) |
|  | Asia | 0.68 (0.52, 0.89) | 0.94 (0.73, 1.23) |
|  | Africa | 0.84 (0.49, 1.42) | 0.91 (0.56, 1.49) |
|  | North America | 0.85 (0.47, 1.53) | 1.09 (0.63, 1.87) |
|  | South America | 1.08 (0.75, 1.55) | 1.05 (0.66, 1.65) |
|  | p-value | 0.012 | 0.182 |
| Cholinesterase inhibitors | Sweden | reference | reference |
|  | Other EU & Nordic countries | 0.84 (0.76, 0.93) | 0.92 (0.81, 1.05) |
|  | The other European countries | 0.68 (0.51, 0.89) | 0.81 (0.60, 1.09) |
|  | Asia | 0.80 (0.56, 1.14) | 1.33 (0.91, 1.95) |
|  | Africa | 0.44 (0.22, 0.86) | 0.46 (0.21, 1.01) |
|  | North America | 0.92 (0.49, 1.75) | 0.84 (0.43, 1.67) |
|  | South America | 1.30 (0.80, 2.12) | 0.93 (0.52, 1.67) |
|  | p-value | < 0.001 | 0.135 |
| Memantine | Sweden | reference | reference |
|  | Other EU & Nordic countries | 0.95 (0.88, 1.04) | 1.00 (0.89, 1.12) |
|  | The other European countries | 1.10 (0.86, 1.40) | 1.21 (0.92, 1.59) |
|  | Asia | 0.66 (0.48, 0.89) | 0.57 (0.42, 0.78) |
|  | Africa | 0.83 (0.44, 1.54) | 0.81 (0.39, 1.68) |
|  | North America | 1.12 (0.63, 1.98) | 0.61 (0.32, 1.16) |
|  | South America | 0.85 (0.57, 1.25) | 0.60 (0.36, 1.01) |
|  | p-value | 0.122 | 0.002 |
| Results were presented as odds ratios (95% confidence interval).  Binary logistic regression model was adjusted for age, living alone, living areas (urban/intermediate/rural), education, income, Charlson Comorbidity Index, year of diagnosis and type of diagnosis unit.  Basic dementia diagnostic work-up refers to the completion of four tests: clock test, blood analysis, Mini-Mental State Examination (MMSE) and computed tomography or magnetic resonance imaging (CT-MRI).  Only persons with Alzheimer’s disease, mixed dementia, Lewy Body dementia or Parkinson’s disease dementia (n = 42,005) were analyzed for Cholinesterase inhibitors and Memantine.  p-values were calculated by Wald test. | | | |

## Supplementary Table 5. Region of birth in association with dementia diagnostics and the use of anti-dementia drugs, stratified by types of diagnostic unit

|  |  | **Primary care (n = 34,762)** | **Memory clinic (n = 43,490)** |
| --- | --- | --- | --- |
| Basic diagnostic work-up | Sweden | reference | reference |
|  | Other EU & Nordic countries | 1.06 (0.97, 1.16) | 0.88 (0.80, 0.96) |
|  | The other European countries | 0.59 (0.47, 0.73) | 0.45 (0.38, 0.54) |
|  | Asia | 0.78 (0.54, 1.11) | 0.40 (0.33, 0.48) |
|  | Africa | 0.78 (0.36, 1.69) | 0.28 (0.20, 0.40) |
|  | North America | 1.04 (0.59, 1.84) | 1.04 (0.62, 1.75) |
|  | South America | 0.85 (0.40, 1.82) | 0.90 (0.63, 1.29) |
|  | p-value | < 0.001 | < 0.001 |
| Clock test | Sweden | reference | reference |
|  | Other EU & Nordic countries | 0.97 (0.87, 1.09) | 0.83 (0.75, 0.93) |
|  | The other European countries | 0.63 (0.49, 0.80) | 0.44 (0.36, 0.54) |
|  | Asia | 0.59 (0.41, 0.86) | 0.43 (0.34, 0.53) |
|  | Africa | 0.44 (0.21, 0.94) | 0.26 (0.18, 0.38) |
|  | North America | 0.71 (0.38, 1.34) | 0.72 (0.42, 1.25) |
|  | South America | 0.75 (0.33, 1.70) | 1.06 (0.68, 1.64) |
|  | p-value | 0.001 | < 0.001 |
| Blood analysis | Sweden | reference | reference |
|  | Other EU & Nordic countries | 0.90 (0.77, 1.06) | 0.99 (0.83, 1.18) |
|  | The other European countries | 0.83 (0.56, 1.21) | 0.60 (0.43, 0.84) |
|  | Asia | 1.03 (0.55, 1.92) | 1.08 (0.68, 1.73) |
|  | Africa | 1.14 (0.27, 4.89) | 1.05 (0.42, 2.59) |
|  | North America | 1.87 (0.45, 7.69) | 0.79 (0.32, 1.95) |
|  | South America | 0.37 (0.14, 0.98) | 1.41 (0.62, 3.19) |
|  | p-value | 0.332 | 0.117 |
| MMSE | Sweden | reference | reference |
|  | Other EU & Nordic countries | 0.83 (0.72, 0.94) | 0.76 (0.65, 0.88) |
|  | The other European countries | 0.39 (0.30, 0.50) | 0.26 (0.21, 0.32) |
|  | Asia | 0.50 (0.34, 0.74) | 0.23 (0.18, 0.29) |
|  | Africa | 0.43 (0.19, 0.97) | 0.25 (0.16, 0.40) |
|  | North America | 0.55 (0.28, 1.11) | 0.78 (0.34, 1.79) |
|  | South America | 0.47 (0.20, 1.07) | 0.67 (0.40, 1.12) |
|  | p-value | < 0.001 | < 0.001 |
| CT-MRI | Sweden | reference | reference |
|  | Other EU & Nordic countries | 1.20 (1.07, 1.33) | 1.17 (0.95, 1.43) |
|  | The other European countries | 0.92 (0.70, 1.20) | 0.88 (0.55, 1.40) |
|  | Asia | 1.62 (0.97, 2.70) | 0.68 (0.41, 1.11) |
|  | Africa | 1.03 (0.38, 2.79) | 0.56 (0.22, 1.41) |
|  | North America | 1.38 (0.71, 2.70) | 1.76 (0.43,7.19) |
|  | South America | 0.79 (0.35, 1.82) | 0.92 (0.43, 1.99) |
|  | p-value | 0.018 | 0.305 |
| Neuropsychological assessment | Sweden | reference | reference |
|  | Other EU & Nordic countries | 0.85 (0.66, 1.11) | 0.85 (0.79, 0.92) |
|  | The other European countries | 0.76 (0.40, 1.47) | 0.31 (0.24, 0.39) |
|  | Asia | 1.13 (0.52, 2.44) | 0.38 (0.31, 0.48) |
|  | Africa | 1.00 (1.00, 1.00) | 0.27 (0.18, 0.42) |
|  | North America | 1.85 (0.44, 7.70) | 0.99 (0.65, 1.50) |
|  | South America | 1.09 (0.14, 8.38) | 0.53 (0.38, 0.75) |
|  | p-value | 0.745 | < 0.001 |
| Occupational therapy assessment | Sweden | reference | reference |
|  | Other EU & Nordic countries | 1.04 (0.95, 1.13) | 1.17 (1.10, 1.25) |
|  | The other European countries | 0.94 (0.74, 1.19) | 0.91 (0.77, 1.07) |
|  | Asia | 0.69 (0.46, 1.03) | 1.20 (1.01, 1.43) |
|  | Africa | 0.89 (0.39, 2.03) | 1.23 (0.88, 1.71) |
|  | North America | 1.23 (0.71, 2.14) | 1.38 (0.95, 2.01) |
|  | South America | 1.57 (0.75, 3.31) | 1.47 (1.12, 1.94) |
|  | p-value | 0.370 | < 0.001 |
| Lumbar puncture | Sweden | reference | reference |
|  | Other EU & Nordic countries | 0.72 (0.56, 0.93) | 0.98 (0.91, 1.05) |
|  | The other European countries | 0.68 (0.34, 1.36) | 0.71 (0.59, 0.84) |
|  | Asia | 0.12 (0.02, 0.88) | 0.82 (0.68, 0.99) |
|  | Africa | 1.29 (0.28, 5.92) | 0.82 (0.57, 1.18) |
|  | North America | 0.83 (0.11, 6.04) | 0.97 (0.65, 1.46) |
|  | South America | 3.02 (0.86, 10.66) | 1.02 (0.76, 1.36) |
|  | p-value | 0.023 | 0.005 |
| Cholinesterase inhibitors | Sweden | reference | reference |
|  | Other EU & Nordic countries | 0.89 (0.75, 1.04) | 0.86 (0.79, 0.95) |
|  | The other European countries | 0.74 (0.48, 1.14) | 0.73 (0.58, 0.92) |
|  | Asia | 0.43 (0.23, 0.80) | 1.19 (0.88, 1.59) |
|  | Africa | 0.35 (0.10, 1.22) | 0.45 (0.26, 0.80) |
|  | North America | 0.91 (0.32, 2.60) | 0.88 (0.52, 1.48) |
|  | South America | 1.82 (0.38, 8.67) | 1.09 (0.74, 1.60) |
|  | p-value | 0.042 | < 0.001 |
| Memantine | Sweden | reference | reference |
|  | Other EU & Nordic countries | 0.99 (0.86, 1.14) | 0.97 (0.89, 1.05) |
|  | The other European countries | 1.06 (0.72, 1.56) | 1.16 (0.95, 1.43) |
|  | Asia | 0.70 (0.39, 1.29) | 0.58 (0.46, 0.74) |
|  | Africa | 0.98 (0.29, 3.34) | 0.80 (0.48, 1.34) |
|  | North America | 1.47 (0.58, 3.74) | 0.75 (0.46, 1.20) |
|  | South America | 0.43 (0.12, 1.62) | 0.78 (0.56, 1.08) |
|  | p-value | 0.725 | < 0.001 |
| Results were presented as odds ratios (95% confidence interval).  Binary logistic regression model was adjusted for age, sex, living alone, living areas (urban/intermediate/rural), education, income, Charlson Comorbidity Index and year of diagnosis.  Basic dementia diagnostic work-up refers to the completion of four tests: clock test, blood analysis, Mini-Mental State Examination (MMSE) and computed tomography or magnetic resonance imaging (CT-MRI).  Only persons with Alzheimer’s disease, mixed dementia, Lewy Body dementia or Parkinson’s disease dementia (n = 42,005) were analyzed for Cholinesterase inhibitors and Memantine.  p-values were calculated by Wald test. | | | |

## Supplementary Table 6. Region of birth in association with dementia diagnostics and the use of anti-dementia drugs, stratified by year of diagnosis

|  |  | **2007-2010 (n = 16,527)** | **2011-2018 (n = 61,725)** |
| --- | --- | --- | --- |
| Basic diagnostic work-up | Sweden | reference | reference |
|  | Other EU & Nordic countries | 0.86 (0.75, 0.98) | 1.03 (0.96, 1.11) |
|  | The other European countries | 0.54 (0.39, 0.74) | 0.50 (0.43, 0.58) |
|  | Asia | 0.77 (0.48, 1.23) | 0.42 (0.35, 0.50) |
|  | Africa | 0.21 (0.09, 0.50) | 0.33 (0.23, 0.47) |
|  | North America | 1.08 (0.53, 2.20) | 1.04 (0.66, 1.63) |
|  | South America | 0.89 (0.48, 1.66) | 0.93 (0.64, 1.34) |
|  | p-value | < 0.001 | < 0.001 |
| Clock test | Sweden | reference | reference |
|  | Other EU & Nordic countries | 0.81 (0.70, 0.94) | 0.95 (0.87, 1.04) |
|  | The other European countries | 0.70 (0.49, 1.01) | 0.48 (0.41, 0.57) |
|  | Asia | 0.91 (0.53, 1.56) | 0.44 (0.36, 0.54) |
|  | Africa | 0.27 (0.12, 0.65) | 0.31 (0.21, 0.45) |
|  | North America | 0.68 (0.33, 1.41) | 0.76 (0.46, 1.27) |
|  | South America | 0.93 (0.45, 1.93) | 1.08 (0.68, 1.70) |
|  | p-value | 0.004 | < 0.001 |
| Blood analysis | Sweden | reference | reference |
|  | Other EU & Nordic countries | 0.97 (0.75, 1.26) | 0.97 (0.85, 1.11) |
|  | The other European countries | 0.96 (0.51, 1.82) | 0.65 (0.50, 0.86) |
|  | Asia | 0.66 (0.31, 1.43) | 1.19 (0.77, 1.83) |
|  | Africa | 1.32 (0.17,9.99) | 1.00 (0.44, 2.30) |
|  | North America | 1.03 (0.25, 4.30) | 1.16 (0.47, 2.85) |
|  | South America | 0.80 (0.25, 2.58) | 1.06 (0.52, 2.17) |
|  | p-value | 0.969 | 0.096 |
| MMSE | Sweden | reference | reference |
|  | Other EU & Nordic countries | 0.73 (0.61, 0.87) | 0.87 (0.77, 0.98) |
|  | The other European countries | 0.30 (0.21, 0.43) | 0.32 (0.26, 0.38) |
|  | Asia | 0.51 (0.29, 0.89) | 0.26 (0.21, 0.33) |
|  | Africa | 0.19 (0.08, 0.46) | 0.33 (0.21, 0.52) |
|  | North America | 2.18 (0.52,9.17) | 0.49 (0.28, 0.85) |
|  | South America | 0.54 (0.25, 1.16) | 0.72 (0.43, 1.22) |
|  | p-value | < 0.001 | < 0.001 |
| CT-MRI | Sweden | reference | reference |
|  | Other EU & Nordic countries | 1.03 (0.84, 1.27) | 1.27 (1.13, 1.41) |
|  | The other European countries | 0.67 (0.42, 1.07) | 0.98 (0.75, 1.28) |
|  | Asia | 1.24 (0.52, 2.97) | 1.05 (0.70, 1.57) |
|  | Africa | 0.53 (0.15, 1.93) | 0.80 (0.35, 1.84) |
|  | North America | 1.07 (0.37, 3.09) | 1.66 (0.81, 3.40) |
|  | South America | 1.25 (0.38, 4.08) | 0.77 (0.41, 1.46) |
|  | p-value | 0.631 | 0.002 |
| Neuropsychological assessment | Sweden | reference | reference |
|  | Other EU & Nordic countries | 0.88 (0.76, 1.01) | 0.84 (0.77, 0.92) |
|  | The other European countries | 0.32 (0.20, 0.50) | 0.34 (0.26, 0.44) |
|  | Asia | 0.62 (0.38, 1.02) | 0.38 (0.30, 0.49) |
|  | Africa | 0.17 (0.05, 0.66) | 0.30 (0.19, 0.46) |
|  | North America | 0.99 (0.47, 2.08) | 1.06 (0.66, 1.70) |
|  | South America | 0.77 (0.40, 1.48) | 0.49 (0.33, 0.72) |
|  | p-value | < 0.001 | < 0.001 |
| Occupational therapy assessment | Sweden | reference | reference |
|  | Other EU & Nordic countries | 1.13 (1.01, 1.27) | 1.16 (1.10, 1.23) |
|  | The other European countries | 0.79 (0.58, 1.08) | 0.95 (0.82, 1.10) |
|  | Asia | 0.76 (0.50, 1.17) | 1.16 (0.99, 1.38) |
|  | Africa | 0.74 (0.31, 1.76) | 1.30 (0.94, 1.80) |
|  | North America | 1.49 (0.81, 2.77) | 1.33 (0.93, 1.90) |
|  | South America | 1.35 (0.78, 2.32) | 1.61 (1.21, 2.15) |
|  | p-value | 0.061 | < 0.001 |
| Lumbar puncture | Sweden | reference | reference |
|  | Other EU & Nordic countries | 0.95 (0.83, 1.08) | 0.97 (0.89, 1.04) |
|  | The other European countries | 0.56 (0.39, 0.81) | 0.76 (0.63, 0.92) |
|  | Asia | 0.67 (0.41, 1.09) | 0.82 (0.67, 1.01) |
|  | Africa | 0.86 (0.33, 2.25) | 0.86 (0.58, 1.26) |
|  | North America | 1.19 (0.59, 2.40) | 0.87 (0.54, 1.42) |
|  | South America | 0.74 (0.40, 1.35) | 1.20 (0.87, 1.65) |
|  | p-value | 0.042 | 0.042 |
| Cholinesterase inhibitors | Sweden | reference | reference |
|  | Other EU & Nordic countries | 0.85 (0.71, 1.01) | 0.87 (0.80, 0.95) |
|  | The other European countries | 0.55 (0.34, 0.92) | 0.77 (0.61, 0.96) |
|  | Asia | 0.94 (0.45, 1.97) | 1.01 (0.76, 1.33) |
|  | Africa | 0.17 (0.05, 0.51) | 0.58 (0.32, 1.05) |
|  | North America | 0.89 (0.37, 2.14) | 0.88 (0.51, 1.54) |
|  | South America | 1.46 (0.61, 3.54) | 1.06 (0.71, 1.61) |
|  | p-value | 0.006 | 0.009 |
| Memantine | Sweden | reference | reference |
|  | Other EU & Nordic countries | 0.97 (0.84, 1.13) | 0.96 (0.89, 1.04) |
|  | The other European countries | 1.08 (0.70, 1.68) | 1.14 (0.93, 1.39) |
|  | Asia | 1.26 (0.69, 2.28) | 0.53 (0.42, 0.68) |
|  | Africa | 0.77 (0.26, 2.29) | 0.86 (0.51, 1.45) |
|  | North America | 0.59 (0.27, 1.33) | 0.99 (0.60, 1.65) |
|  | South America | 0.55 (0.27, 1.12) | 0.83 (0.58, 1.18) |
|  | p-value | 0.491 | < 0.001 |
| Results were presented as odds ratios (95% confidence interval).  Binary logistic regression model was adjusted for age, sex, living alone, living areas (urban/intermediate/rural), education, income, Charlson Comorbidity Index and type of diagnostic unit.  Basic dementia diagnostic work-up refers to the completion of four tests: clock test, blood analysis, Mini-Mental State Examination (MMSE) and computed tomography or magnetic resonance imaging (CT-MRI).  Only persons with Alzheimer’s disease, mixed dementia, Lewy Body dementia or Parkinson’s disease dementia (n = 42,005) were analyzed for Cholinesterase inhibitors and Memantine.  p-values were calculated by Wald test. | | | |

## Supplementary Table 7. Region of birth in association with dementia diagnostics and the use of anti-dementia drugs, additionally adjusted by MMSE score at baseline

|  |  | **Model 1 (n = 73,211)** | **Model 2 (n = 78,252)** |
| --- | --- | --- | --- |
| Clock test | Sweden | reference | reference |
|  | Other EU & Nordic countries | 1.05 (0.96, 1.15) | 1.10 (1.02, 1.20) |
|  | The other European countries | 0.97 (0.78, 1.21) | 1.00 (0.85, 1.19) |
|  | Asia | 0.80 (0.62, 1.04) | 0.88 (0.72, 1.08) |
|  | Africa | 0.44 (0.29, 0.67) | 0.62 (0.43, 0.90) |
|  | North America | 0.73 (0.45, 1.18) | 0.75 (0.48, 1.17) |
|  | South America | 1.34 (0.83, 2.16) | 1.56 (1.04, 2.35) |
|  | p-value | 0.001 | 0.002 |
| Blood analysis | Sweden | reference | reference |
|  | Other EU & Nordic countries | 0.98 (0.86, 1.12) | 1.05 (0.93, 1.18) |
|  | The other European countries | 0.89 (0.65, 1.23) | 0.93 (0.72, 1.20) |
|  | Asia | 1.25 (0.79, 1.99) | 1.41 (0.97, 2.05) |
|  | Africa | 0.96 (0.42, 2.21) | 1.50 (0.69, 3.23) |
|  | North America | 1.24 (0.51, 3.03) | 1.16 (0.54, 2.50) |
|  | South America | 0.89 (0.47, 1.70) | 1.17 (0.63, 2.15) |
|  | p-value | 0.927 | 0.496 |
| CT-MRI | Sweden | reference | reference |
|  | Other EU & Nordic countries | 1.29 (1.16, 1.43) | 1.30 (1.18, 1.43) |
|  | The other European countries | 1.11 (0.84, 1.48) | 1.18 (0.93, 1.49) |
|  | Asia | 1.28 (0.82, 1.98) | 1.45 (1.01, 2.08) |
|  | Africa | 0.77 (0.34, 1.75) | 1.00 (0.50, 2.00) |
|  | North America | 1.72 (0.85, 3.48) | 1.48 (0.81, 2.69) |
|  | South America | 0.87 (0.47, 1.61) | 1.05 (0.60, 1.84) |
|  | p-value | < 0.001 | < 0.001 |
| Neuropsychological assessment | Sweden | reference | reference |
|  | Other EU & Nordic countries | 1.07 (0.99, 1.16) | 1.02 (0.95, 1.11) |
|  | The other European countries | 0.63 (0.49, 0.81) | 0.59 (0.47, 0.75) |
|  | Asia | 0.80 (0.62, 1.03) | 0.75 (0.59, 0.95) |
|  | Africa | 0.75 (0.46, 1.23) | 0.59 (0.37, 0.94) |
|  | North America | 1.22 (0.77, 1.91) | 1.24 (0.81, 1.91) |
|  | South America | 0.89 (0.63, 1.27) | 0.78 (0.55, 1.10) |
|  | p-value | 0.002 | < 0.001 |
| Occupational therapy assessment | Sweden | reference | reference |
|  | Other EU & Nordic countries | 1.15 (1.09, 1.21) | 1.15 (1.10, 1.22) |
|  | The other European countries | 0.91 (0.79, 1.05) | 0.91 (0.80, 1.04) |
|  | Asia | 0.99 (0.84, 1.18) | 1.08 (0.93, 1.27) |
|  | Africa | 1.00 (0.72, 1.40) | 1.18 (0.88, 1.60) |
|  | North America | 1.30 (0.94, 1.79) | 1.35 (1.00, 1.84) |
|  | South America | 1.56 (1.19, 2.03) | 1.55 (1.20, 2.00) |
|  | p-value | < 0.001 | < 0.001 |
| Lumbar puncture | Sweden | reference | reference |
|  | Other EU & Nordic countries | 1.03 (0.96, 1.11) | 1.03 (0.96, 1.10) |
|  | The other European countries | 0.93 (0.77, 1.12) | 0.91 (0.76, 1.08) |
|  | Asia | 1.00 (0.82, 1.23) | 1.02 (0.84, 1.23) |
|  | Africa | 1.11 (0.74, 1.65) | 1.16 (0.81, 1.67) |
|  | North America | 1.02 (0.68, 1.54) | 1.03 (0.69, 1.53) |
|  | South America | 1.14 (0.85, 1.53) | 1.20 (0.90, 1.60) |
|  | p-value | 0.865 | 0.648 |
| Cholinesterase inhibitors | Sweden | reference | reference |
|  | Other EU & Nordic countries | 0.97 (0.90, 1.06) | 0.96 (0.89, 1.04) |
|  | The other European countries | 1.06 (0.85, 1.33) | 1.03 (0.84, 1.27) |
|  | Asia | 1.57 (1.16, 2.12) | 1.44 (1.10, 1.88) |
|  | Africa | 0.69 (0.37, 1.26) | 0.69 (0.41, 1.18) |
|  | North America | 0.99 (0.60, 1.62) | 0.93 (0.58, 1.51) |
|  | South America | 1.25 (0.84, 1.85) | 1.35 (0.93, 1.97) |
|  | p-value | 0.055 | 0.045 |
| Memantine | Sweden | reference | reference |
|  | Other EU & Nordic countries | 0.94 (0.88, 1.01) | 0.95 (0.88, 1.01) |
|  | The other European countries | 1.11 (0.91, 1.35) | 1.06 (0.88, 1.27) |
|  | Asia | 0.54 (0.43, 0.69) | 0.56 (0.45, 0.69) |
|  | Africa | 0.80 (0.47, 1.36) | 0.75 (0.47, 1.19) |
|  | North America | 0.80 (0.52, 1.24) | 0.83 (0.55, 1.28) |
|  | South America | 0.71 (0.51, 0.98) | 0.71 (0.52, 0.97) |
|  | p-value | < 0.001 | < 0.001 |
| Results were presented as odds ratios (95% confidence interval).  Binary logistic regression model was adjusted for age, sex, living alone, living areas (urban/intermediate/rural), education, income, Charlson Comorbidity Index, year of diagnosis, type of diagnosis unit and Mini-Mental State Examination score.  Model 1 was performed on people who received Mini-Mental State Examination.  Model 2 was performed on people who both received and did not receive Mini-Mental State Examination. They did not receive Mini-Mental State Examination because they presumably had severe dementia. We assumed that their scores were 5 points.  Only persons with Alzheimer’s disease, mixed dementia, Lewy Body dementia or Parkinson’s disease dementia were analyzed for Cholinesterase inhibitors and Memantine.  p-values were calculated by Wald test. | | | |

## References

(1) Benchimol EI, Smeeth L, Guttmann A, Harron K, Moher D, Petersen I, Sørensen HT, von Elm E, Langan SM (2015) The REporting of studies Conducted using Observational Routinely-collected health Data (RECORD) statement. *PLoS Med* **12**, e1001885.

(2) World Health Organization (2015) *International statistical classification of diseases and related health problems*, World Health Organization, Geneva.

(3) World Health Organization Collaborating Centre for Drug Statistics Methodology, ATC/DDD Index 2021, <https://www.whocc.no/atc_ddd_index/>, Last updated October 22, 2021, Accessed on October 22, 2021.
